# Ameliorative Effects of Vitamins A, C, and E on Sperm Parameters, Testis Histopathology, and Oxidative Stress Status in Zinc Oxide Nanoparticle-Treated Rats

**DOI:** 10.1155/2023/4371611

**Published:** 2023-01-17

**Authors:** Nasrin Ziamajidi, Maryam Khajvand-Abedini, Sajedeh Daei, Roghayeh Abbasalipourkabir, Alireza Nourian

**Affiliations:** ^1^Department of Clinical Biochemistry, School of Medicine, Hamadan University of Medical Sciences, Hamadan, Iran; ^2^Molecular Medicine Research Center, Hamadan University of Medical Sciences, Hamadan, Iran; ^3^Department of Pathobiology, Faculty of Veterinary Science, Bu-Ali Sina University, Hamadan, Iran

## Abstract

One of the most often utilized nanoparticles (NPs) in several technologies is zinc oxide (ZnO) NPs. However, these NPs are said to have harmful effects on the reproductive system. Thus, we designed this study to specify the potential preventive activity of vitamins (Vits) A, C, and E, as antioxidants, against toxicity of ZnO NPs in the testes of rats. A total of 54 Wistar rats were arranged in 9 groups of 6 and then orally received water (control 1), olive oil (control 2), Vit A (1000 IU/kg), Vit C (200 mg/kg), Vit E (100 IU/kg), ZnO (200 mg/kg), ZnO+Vit A, ZnO+Vit C, and ZnO+Vit E. To determine the amount of testicular injury, sperm analysis and histological evaluation were performed. In addition, oxidative stress status was examined using colorimetric and qRT-PCR methods. Our findings suggest that ZnO NPs cause adverse effects on sperm parameters and testicular histology. Furthermore, oxidative biomarkers (malondialdehyde and total oxidant capacity) were enhanced in the ZnO group. By contrast, the gene expression and activities of antioxidant enzymes (SOD, GPx, and CAT) noted a remarkable decrease in the ZnO group regarding control (*p* < 0.05). However, oxidative markers were remarkably mitigated after combined treatment of ZnO NPs and Vits A, C, or E compared to the rats given ZnO NPs (*p* < 0.05). Additionally, compared to the ZnO NP group, the rats receiving Vits+ZnO NPs exhibit increased antioxidant enzyme activity and mRNA expression (*p* < 0.05). The findings demonstrate the abovementioned Vits' ameliorative effects on toxicity incurred by ZnO NPs.

## 1. Introduction

Recent advances in nanotechnology have led to an ever-increasing usage of nanoparticles (NPs) in clinical, medicine, and even industry. This issue has caused serious concerns about their biochemical process within the body and possible toxic effects on living organisms, e.g., humans [1]. One of the widely used NPs is zinc oxide nanoparticles (ZnO NPs) which show distinctive physiochemical and antibacterial characteristics. Due to this, they have been found in numerous products including food preservatives, sunscreens in cosmetics, textile coatings, agricultural fertilizers, and several biomedical applications [2]. Numerous in vivo studies [3–6] have shown the harmful effects of ZnO NPs on many organs. Additionally, it has been shown that nanoparticles may cross the blood-testis barrier (BTB) and harm the testis [2]. The male reproductive system has a crucial defensive mechanism called BTB that may stop foreign particles from penetrating [7]. Although it is theoretically impossible for nanoparticles to get through the BTB, several studies have shown that they can do so and end up being stored in the testes. For example, it was found that small-sized silver nanoparticles (AgNPs) (22 and 42 nm) exist in mice testes after oral administration while the larger ones (71 and 323 nm) are not observed [8]. In this regard, the results of many animal and clinical studies showed that enhanced concentrations of nanoparticles have a direct relationship with reduced quality or number of sperms [9, 10].

The exact route of ZnO NP-induced toxicity, including the dysfunction of the male reproductive system, is still unclear. However, oxidative stress is recognized as a possible mechanism pathway [11, 12]. In this way, it was shown that overproduction of reactive oxygen species (ROS) leads to impaired spermatogenesis and therefore cell apoptosis. Furthermore, previous studies showed that both oxidative stress and changes in antioxidant enzymes result in functional disorders in the reproductive and sexual systems [13].

ROS plays a variety of physiological roles in sperm while the adverse effects of its excessive production have also been revealed. The body uses both enzymatic and nonenzymatic antioxidant defense mechanisms to combat excessive ROS. Oxidative stress may be reduced by enzymatic antioxidant systems like catalase (CAT), superoxide dismutase (SOD), and glutathione peroxidase (GPx). Additionally, a number of studies have shown that oral vitamin (Vit) delivery, which acts as a nonenzymatic antioxidant, may raise sperm survival rates, decrease the number of immobile sperm, and improve sperm motility in infertile men [14–17]. Vit A is a vital dietary compound that is required to provide normal responses under stress and illness conditions [17]. Vit E (*α*-tocopherol) is a lipid-soluble antioxidant with free oxygen radical-scavenging activity and preserves cell membranes against peroxidative damage [18]. Vit C (ascorbate) helps increase the immunocompetence by adjusting functional enzymes [19].

According to previous research, ZnO NPs cause notable male reproductive toxicity [20]. Therefore, the goal of this work is to offer insight on how Vits A, C, and E affect toxicity caused by ZnO NPs in rat testes.

## 2. Materials and Methods

### 2.1. Chemicals

ZnO NPs with the approximate size of 20 nm and spherical shape were obtained from Nanosany Company (Mashhad, Iran). Some properties of ZnO NPs were reported in the prior work [21].

Vit A, C, and E supplementations including all-transretinoic acid (≤98%), L-ascorbic acid (99%), and *α*-tocopherol (≤96%) were bought from Sigma-Aldrich Co. (St. Louis, MO, USA).

Vit A and Vit E were dispersed in olive oil to achieve an appropriate dose of Vit A (1000 IU/kg body weight) and Vit E (100 IU/kg body weight) according to the previous studies [22, 23]. Moreover, the suggested dose of Vit C (200 mg/kg body weight) was achieved by using distilled water [24].

### 2.2. Ethical Statement

In this research, all of the protocols were carried out with regard to the ethical guidelines of the Institutional Animal Ethics Committee of Hamadan University of Medical Sciences (IR.UMSHA.REC.1400.268).

### 2.3. Preparing ZnO NP suspension

The concentration 2 g/ml of ZnO NPs was prepared in bidistilled water as a stock solution and kept at 4°C. The considered concentration of NPs was 200 mg/kg body weight [25] which was obtained by bidistilled water and employed to oral administration (gavage) of animals at the earliest possible moment.

### 2.4. Animals and Treatment

In this research, 54 adult male Wistar rats at the age of 7-8 weeks and weights between 150 and 200 g were acquired from Hamadan University of Medical Sciences (Hamadan, Iran). Standard ambient of laboratory (relative humidity of 50 ± 5%, temperature of 25 ± 2°C, and 12 h light-dark cycles) was kept until the end of the experiment.

The experimental rats were randomly distributed into 9 groups each of which containing 6 rats. It should be noted that Vit therapy was started one week before the administration of ZnO NPs. The treatment period lasted 3 weeks and was done by oral administration. A list of experimental groups of rats is presented below.

1. Control 1: 1 ml/day bidistilled water was given
2. Control 2: 1 ml/day olive oil was given
3. Vit A: Vit A (1000 IU/kg) was given
4. Vit C: Vit C (200 mg/kg) was given
5. Vit E: Vit E (100 IU/kg) was given
6. ZnO: ZnO NPs (200 mg/kg) were given
7. ZnO+Vit A: ZnO NPs (200 mg/kg) and Vit A (1000 IU/kg) were given
8. ZnO+Vit C: ZnO NPs (200 mg/kg) and Vit C (200 mg/kg) were given
9. ZnO+Vit E: ZnO NPs (200 mg/kg) and Vit E (100 IU/kg) were given

After completion of 24 h from the last administration, the animals were weighed and then sacrificed. Afterwards, the testes of each rat were immediately dissected out and weighed.

### 2.5. Sperm Analysis

Briefly, the left cauda epididymis from each rat was carefully taken off and transferred into a Petri dish that contained fresh Ham's F10 medium. The sperm count was estimated using a hemocytometer following the World Health Organization (WHO) recommendations. In order to explain in more detail, the diluted sperm suspension (1 ml of sperm suspension was diluted in 9 ml normal saline solution 3%) was transferred to each counting side of a hemocytometer. Then, the settled sperms were counted with a light microscope (Zeiss, Munich, Germany). Furthermore, we evaluated the morphology and motility of sperms with the aid of a light microscope. The viability was also assessed with eosin dye.

### 2.6. Testis Histopathological and Histomorphometry Assessments

Immediately after euthanasia, the right testicle of all experimental rats was removed and placed in 10% neutral buffered formalin for 24 h. After dehydration in ascending ethanol series, the samples were cleared in xylene, infiltrated and embedded in paraffin, and sectioned at 5-6 *μ*m thickness using a rotary microtome (Leica RM2255, Germany). The sections were then stained with hematoxylin and eosin (H&E) and observed by a veterinary pathologist with the aid of a light microscope (Olympus CX41, Japan) equipped with a digital camera (Olympus DP25, Germany).

The atrophy of seminiferous tubules was assessed quantitatively by measuring the tubular surface area. For each group, a number of 20 seminiferous tubules with round to slightly oval shape were selected in different microscopic fields. The area was measured by outlining tubular periphery using Olympus DP2-BSW application software (v2.2). All images were taken as JPEG pictures at ×100 magnification from H&E-stained testicular tissue sections.

### 2.7. Biochemical Parameters

#### 2.7.1. Measuring Testis Oxidant/Antioxidant Levels

First, the preparation of testis lysates was performed as regards to the Kiazist kit protocol (Hamedan, Iran). Then, the content of malondialdehyde (MDA), total oxidant species (TOS), and total antioxidant capacity (TAC) was determined following Kiazist kit manual. Lastly, we calculated the oxidative stress index (OSI) according to the below formula:

(1) $$\begin{array}{c} \text{OSI}\left(\frac{\text{Arbitrary}}{\text{scale}}\right)=\frac{\text{TOS}}{\text{TAS}}. \end{array}$$

#### 2.7.2. Measuring Antioxidant Enzyme Activity

In order to evaluate antioxidant defense system, the activity of antioxidant enzymes including SOD, GPx, and CAT was measured by using colorimetric assay kits following the Kiazist Kit guidelines. Also, protease inhibitor cocktail (Kiazist, KPIMM) was added to lysates for doing this task.

#### 2.7.3. Measuring Total Protein

In order to normalize the oxidative stress parameters, we used the Bradford assay. In this method, standard curve of bovine serum albumin (BSA) was drawn to determine the amount of protein [26].

### 2.8. RNA Isolation and Quantitative Reverse Transcription-Polymerase Chain Reaction (qRT-PCR)

In a nutshell, Kiazol reagent (Kiazist, Iran) was used to isolate the total RNA of the testis tissues following the protocol of company. The purity and concentration of the extracted RNA were evaluated by the 260/280 nm absorbance ratio and NanoDrop One UV-Vis Spectrophotometer (Thermo Fisher Scientific, Waltham, MA), respectively. Also, %1 gel agarose electrophoresis was used to determine the quality of RNA.

The RNA samples were reverse-transcribed into single-stranded cDNA using BioFact™ RT Series cDNA synthesis kit, and a LightCycler®96 instrument (Roche, Germany) was employed to run qRT-PCR assay using a Syber Green qPCR-based method.

Primer3 software was exploited to design the primer sequence for the qRT-PCR reaction (Table 1). The qRT-PCR results were analyzed by the 2^−*ΔΔ*Ct^ method [27], and the relative expression of SOD, GPx, and CAT genes was determined according to the *β*-actin gene expression as an internal control.

### 2.9. Statistical Analysis

All data were analyzed by employing GraphPad prism 9 software, and the results are given as mean ± standard error of mean (SEM). One-way analysis of variance (ANOVA) followed by post hoc Tukey's test was exploited to compare statistical differences between the control and the experimental groups. The statistical significance for differences was considered as the probability (*p*) values less than 0.05.

## 3. Results

### 3.1. Weight of Testis

The weights of testis tissues and relative testis weight (testis weight/body weight) in rats are shown in Table 2. The testis weight noteworthily decreased in the rats given ZnO NPs in comparison to the control (*p* = 0.04), while there were no meaningful changes between other groups (*p* > 0.05). Also, the relative testis weights were not notably changed among different groups (*p* > 0.05).

### 3.2. Sperm Parameters

The evaluation of sperm parameters including sperm count, normal morphology, viability, and motility noted a remarkable decline in ZnO NP-exposed rats regarding the control with a significant level of *p* = 0.014, *p* = 0.014, *p* = 0.003, and *p* = 0.012, respectively (Table 3). The aforementioned parameters of sperm were not remarkably changed in Vits A, C, and E+ZnO-treated rats regarding the control (*p* > 0.05) (Table 3). Thus, oral administrations of Vits A, C, and E could compensate the ZnO NP toxicity. Also, the results indicated that the percentage of sperm parameters did not have meaningful differences between the Vits and control groups (*p* > 0.05).

### 3.3. Histopathological and Histomorphometry Findings

**Figure 1:**
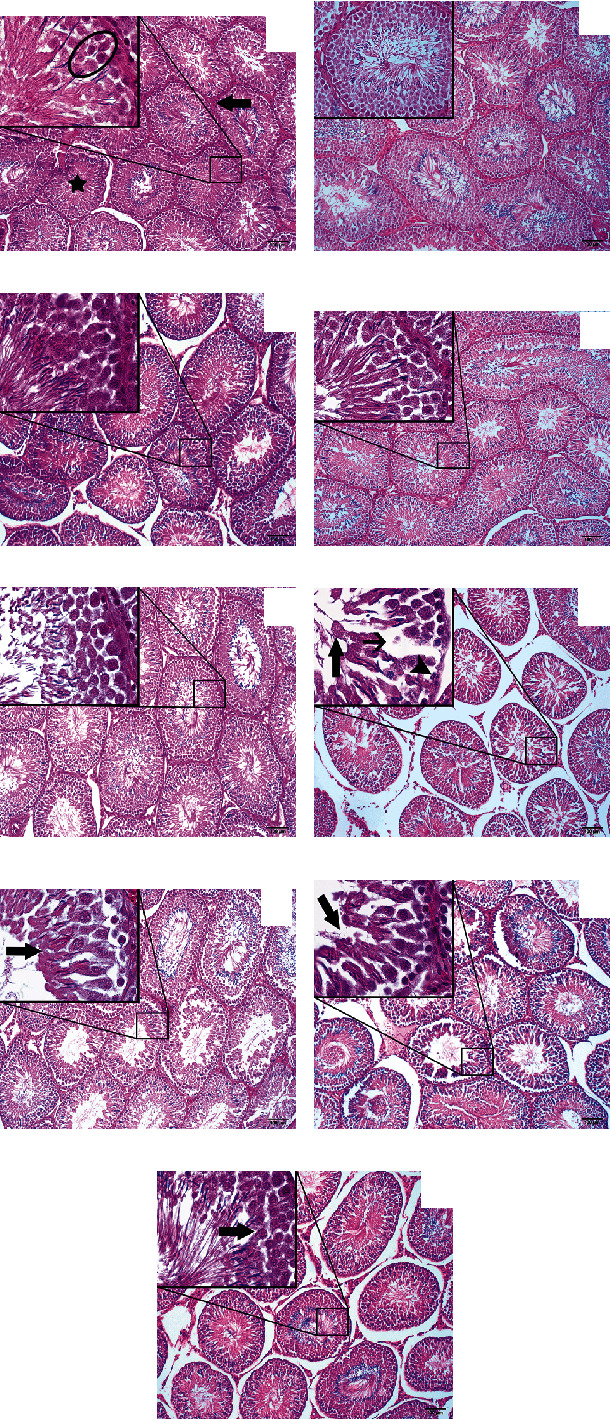
Micrographs of testicular tissue from control and treated rats. (a) Control 1 (Bi-distilled water), (b) control 2 (olive oil), (c) Vit A, (d) Vit C, and (e) Vit E show normal histology of the organ showing regular interstitial connective tissue (large arrow) and seminiferous tubules (asterisk) with typical arrangement of spermatogenic cells in various stages of maturation (ellipse). (f) ZnO NP-treated rats (arrowheads, large arrow and small arrow show atrophy of tubular epithelium, sloughing of Sertoli cells, and intratubular empty spaces, respectively.) (g), (h) and (i), respectively, indicate Vit A+ZnO, Vit C+ZnO, and Vit E+ZnO-treated rats so that the disorganized germinal epithelium of seminiferous tubules (arrow) has partly been improved (×100 magnification (inset ×1000), hematoxylin and eosin).

Histopathological examination indicated normal microscopic regular connective tissue in the control and Vit groups (Figures 1(a)–1(e)). In contrast, in the ZnO NP-treated group, the cytoarchitecture of seminiferous tubules was remarkably altered. Also, the tubular epithelium showed atrophy and degeneration along with apoptosis of spermatogonial cells (arrowheads) next to the peritubular tissue and sloughing of Sertoli cells (large arrow). The depletion of germ cells led to the formation of intratubular empty spaces (small arrow) (see Figure 1(f)). Furthermore, the disorganized germinal epithelium of seminiferous tubules (arrow) slightly regenerated after exposure to Vit A. Also, intratubular empty spaces are being filled with normal tissue in rats exposed to ZnO+Vit A (Figure 1(g)). However, the impaired epithelium of seminiferous tubules (arrow) was not noticeably restored in ZnO+Vit C-treated rats (Figure 1(h)). As it is shown in Figure 1(i), the damaged epithelium of seminiferous tubules (arrow) greatly regenerated, and the tubular lumen is filled with spermatogenic cells and spermatozoa in the ZnO+Vit E group.

**Figure 2:**
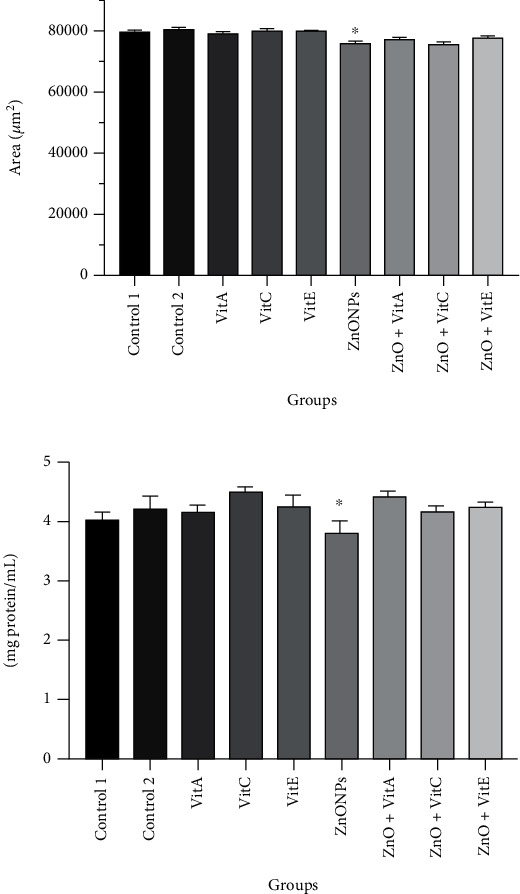
(a) Effects of zinc oxide nanoparticle (ZnO NP) exposure and administration of vitamins A, C, and E on the area of seminiferous tubules (*μ*m^2^). (b) The amount of total protein in tissue lysates is adopted from the Bradford assay. ^∗^*p* < 0.05 shows significant differences compared to the control group. Control 1: distilled water; control 2: olive oil; Vit: vitamin.

As it is shown in Figure 2(a), the area of seminiferous tubules decreased in the ZnO NP group with respect to the control (*p* = 0.002). In contrast, Vit A, C, and E supplementation increased the area so that they became close to the control values.

### 3.4. Protein Concentration

According to the Bradford results, the protein content was lower in the ZnO group compared to the control (*p* < 0.05). However, there were no significant differences between other groups (Figure 2(b)).

### 3.5. Oxidant/Antioxidant Biomarkers

**Figure 3:**
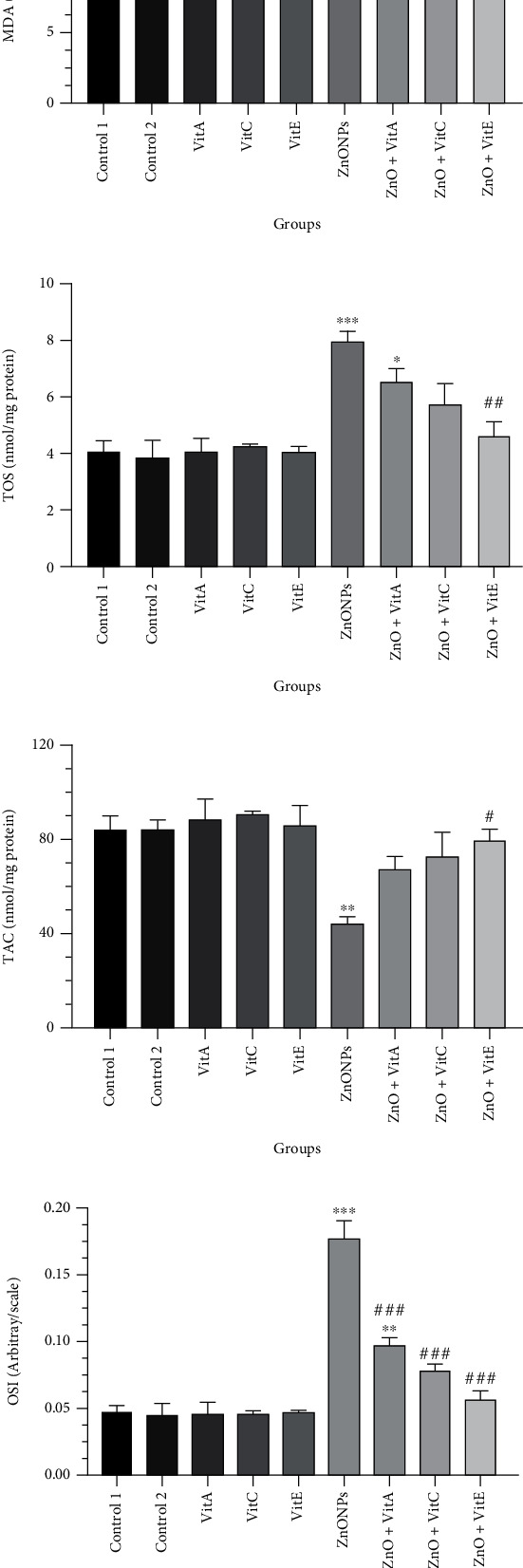
Effects of zinc oxide nanoparticle (ZnO NP) exposure and administration of vitamins A, C, and E on oxidative stress biomarkers in the testis of Wistar rats. (a) Malondialdehyde (MDA), (b) total oxidant status (TOS), (c) total antioxidant capacity (TAC), and (d) oxidative stress index (OSI). The results are expressed as mean ± SEM. ^∗^*p* < 0.05, ^∗∗^*p* < 0.01, and ^∗∗∗^*p* < 0.001 show significant differences compared to the control group, and ^#^*p* < 0.05 and ^###^*p* < 0.001 remarkably altered from the ZnO NP group. Control 1: distilled water; control 2: olive oil; Vit: vitamin.

It can be observed from Figure 3(a) that the MDA level was remarkably increased in rats given ZnO NPs in comparison to the control (*p* = 0.004). By contrast, Vit A, C, and E intake reduced MDA as there were no meaningful differences in Vit A, C, and E+ZnO NP groups and the control one (*p* > 0.05). Like MDA, TOS index was significantly elevated in animals that got only ZnO NPs in comparison to the control (*p* = 0.0002), whereas it was markedly declined in Vit E+ZnO-treated rats compared to the rats that were gavaged with ZnO NPs only (*p* = 0.001) (Figure 3(b)).

As shown in Figure 3(c), the level of TAC was markedly diminished in the ZnO group with respect to the control (*p* < 0.005). Vit A, C, and E intake ameliorated the effects of ZnO NPs by which the TAC level was augmented in the Vit A, C, and E+ZnO group with respect to the ZnO NP group, though this enhancement was not statistically significant in administrations of Vit C and Vit A (*p* > 0.05).

Figure 3(d) shows the OSI level in the experimental groups. Exposure to ZnO NPs caused significant increase in OSI index as compared to the control (*p* < 0.0001). The consumption of Vits could satisfactorily mitigate the increased level of OSI compared to the rats that merely got ZnO NPs (*p* < 0.0001).

### 3.6. Antioxidant Enzyme Activity

**Figure 4:**
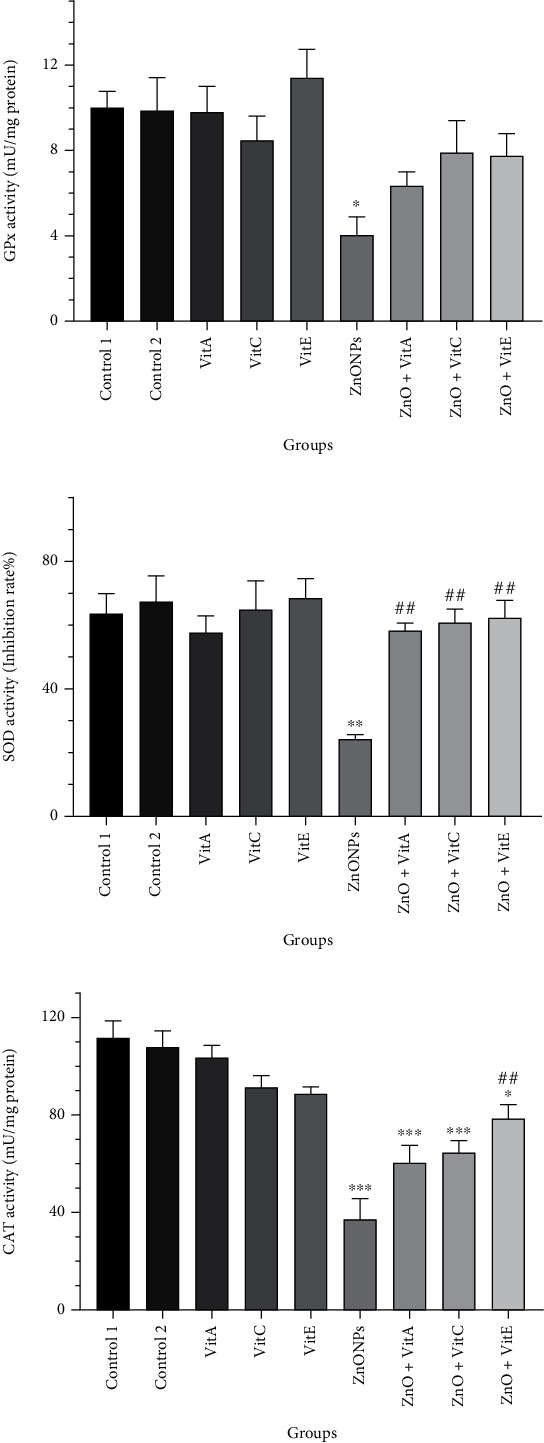
Impacts of zinc oxide nanoparticles (ZnO NPs) and vitamin A, C, and E treatment on antioxidant enzyme activities in the testis of Wistar rats. (a) Glutathione peroxidase (GPx), (b) superoxide dismutase (SOD), and (c) catalase (CAT). The results are expressed as mean ± SEM. ^∗^*p* < 0.05, ^∗∗^*p* < 0.01, and ^∗∗∗^*p* < 0.001 show significant differences compared to the control groups, and ^#^*p* < 0.05 and ^##^*p* < 0.01 significantly changed from the ZnO NP-treated group. Control 1: distilled water; control 2: olive oil; Vit: vitamin.

As it is shown in Figure 4(a), GPx activity was markedly lower in the ZnO NP group than the control one (*p* = 0.02). By contrast, treatment with Vits A, C, and E could ameliorate this reduction; however, it was not statistically significant (*p* > 0.05).

The activity of SOD was meaningfully diminished in the animals that got ZnO NPs (*p* = 0.001), whereas the administration of Vits A, C, and E could significantly elevate the SOD activity in comparison to the ZnO NP one (*p* ≤ 0.007) (Figure 4(b)).

Like GPx and SOD, the CAT activity was notably declined in the rats that only got ZnO NPs with respect to the control (*p* < 0.0001). Meanwhile, the supplementation of Vit E could ameliorate the ZnO NP toxicity in which the CAT activity was remarkably enhanced compared with the ZnO NP group (*p* = 0.001) (Figure 4(c)).

### 3.7. Gene Expression Changes

**Figure 5:**
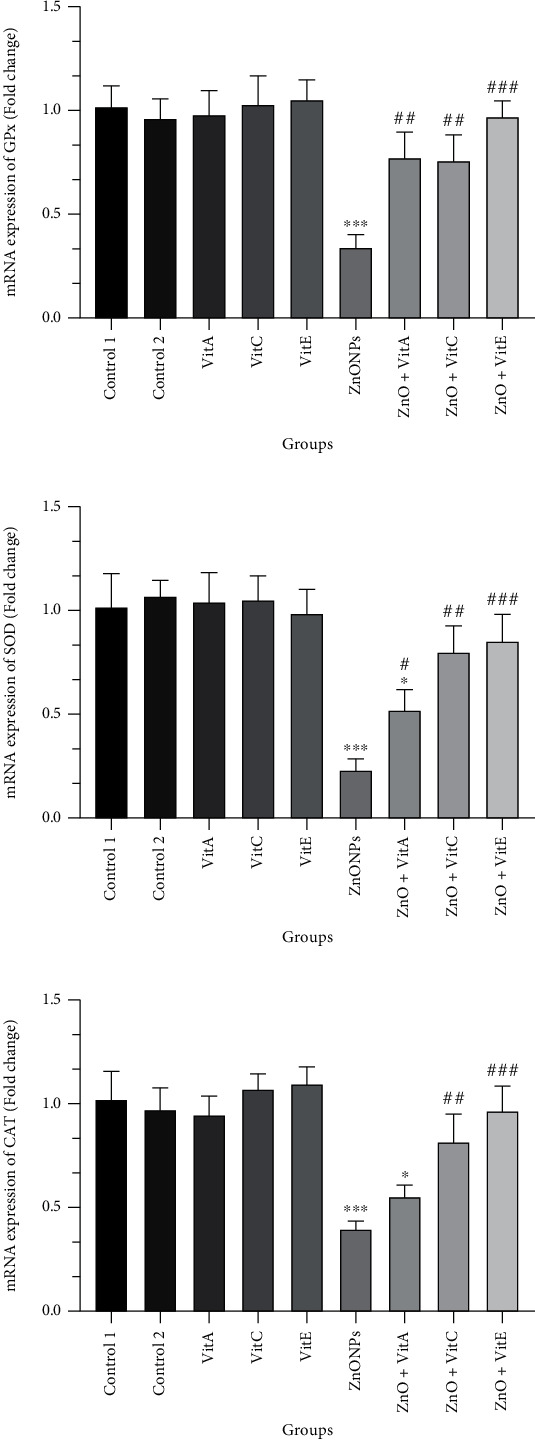
Changes in the mRNA expression of antioxidant-related genes including (a) glutathione peroxidase (GPx), (b) superoxide dismutase (SOD), and (c) catalase (CAT) in the testis of Wistar rats, obtained from reverse transcription-polymerase chain reaction (qRT-PCR) analysis. Method of 2^−*ΔΔ*Ct^ was used to analyze the data in which the *β*-actin gene expression was selected as an endogenous control. The results are reported as fold change compared to the control and expressed as mean ± SEM. ^∗^*p* < 0.05 and ^∗∗∗^*p* < 0.001 show significant differences compared to the control group, and ^#^*p* < 0.05, ^##^*p* < 0.01, and ^###^*p* < 0.001 indicate meaningful differences relative to the ZnO NP-treated group. Control 1: distilled water; control 2: olive oil; Vit: vitamin.

The mRNA expression of antioxidant-related genes in testicular tissue is presented in Figure 5. The data indicated that the gene expression of SOD, GPx, and CAT significantly declined in rats that only got ZnO NPs regarding the control rats (*p* < 0.001). In comparison to ZnO NP-administrated rats, intake of Vits could prevent the significant downregulation of aforementioned genes in the ZnO+Vit A, E, and C groups. However, the gene expression level in the Vit A, C, and E+ZnO groups could not get to the control index.

It is worth mentioning that the activity and gene expression level of GPx, SOD, and CAT were not significantly changed between Vits A, C, E, and the control groups (*p* >0.05).

According to the obtained data, Vit E has the greatest ameliorative effects on ZnO NP-incurred oxidative damage in comparison to Vit A and Vit C.

## 4. Discussion

Reproductive organ dysfunction caused by various nanoparticles has recently received much attention in in vivo studies [20, 28, 29]. Nevertheless, it has not been yet clear how to alleviate the toxic influence of nanoparticles in testis tissue. To the best of our knowledge, the present research is the first to evaluate the ameliorative potential of Vits A, C, and E on ZnO NP-related toxicity in testis tissue. The current study's findings indicate that the testis weight was noticeably reduced in ZnO NP-treated rats in comparison to the control. Vits A, C, and E may significantly offset these alterations, however. Similar to our results, Kuang et al. [20] found that although there were no variations in the relative testis weights of mice, the absolute testis weight was lowered in animals exposed to ZnO NPs. In line with our results, Momeni et al. [30] stated that Vit E could mitigate the induced toxicity in testis tissue as the body and testis weight were improved in the rats exposed to Vit E+sodium arsenite in comparison to the sodium arsenite-treated rats. Moreover, sperm analysis showed adverse effects of ZnO NPs on sperm parameters, especially sperm count. For instance, rats treated with ZnO NPs had significantly less sperm than the control group. Consumption of Vits A, C, and E, however, was able to significantly reverse the ZnO NP-induced alterations since these groups had much more sperm than those only given ZnO NPs. In such a situation, it was also shown that oral exposure to TiO_2_ NPs may lower sperm viability, motility, and count. However, the intake of Vit A and Vit E could mitigate the adverse effects of TiO2 NPs which is fully compliant with our findings regarding the Vit consumption [28]. In this direction, Mumtaz et al. reported that Vit C and Vit E have therapeutic potential against lead toxicity in reproductive organs [31]. Thus, it sounds like that the administration of Vits can be effective to protect sperm from damages incurred by harmful agents.

Besides, the results showed that exposure to ZnO NPs may cause rats' testicles to alter histologically. Vit intake, particularly Vit E, can lessen the negative effects on testicular tissue. Additionally, Abo-Elmaaty et al. [32] demonstrated that Vit E modifies the renal histological changes brought on by cisplatin, which is consistent with our findings.

Regarding the present research, administration of ZnO NPs induces oxidative stress in testis tissue as revealed by a significant enhancement in oxidative parameters. Furthermore, a remarkable reduction was observed in GPx, SOD, and CAT activities and TAC level. Moreover, many research works stated that the toxicity of ZnO NPs is generally caused by excessive ROS production [33, 34], though the defense system is used to attenuate the accumulation of ROS. The first line of the antioxidant defense system includes detoxification enzymes, such as SOD, GPx, and CAT. Additionally, antioxidants in the second line of defense, such as Vits, are crucial in halting the production of free radicals or reactive oxygen species (ROS) [16]. This is consistent with our results, which show that consuming Vits dramatically reduces oxidative stress. It is significant to highlight that in our findings, Vit E had the most protective impact against oxidative stress. Our findings are also endorsed by the histopathological analysis. In former studies, exposure to ZnO and TiO_2_ NPs caused substantial changes in the activity of antioxidant enzymes and heightened MDA content, which resulted in an imbalance in the testis redox system. These observations are consistent with the current study results [29, 35]. In fact, NPs indirectly induce oxidative stress in the testis yielding various cellular alterations both at mitochondrial and nuclear levels. Consequently, peroxidative damage might be induced by an increased level of oxidative stress leading to structural and functional defects of sperm membrane and DNA [36].

The expression of several genes associated to oxidative stress may have changed as a result of ZnO NPs' effects on the testis. As a result, we looked at the expression of the SOD, GPx, and CAT genes in the testes of the experimental groups. According to the qRT-PCR data, rats treated with ZnO NPs had significantly less SOD, GPx, and CAT gene expressions. These alterations were greatly modulated after oral gavage of Vits A, C, and E. In a former report, it was stated that Vits C and E, as antioxidants, have been preferred for their substantial role in relieving the toxicity caused by various pesticides [37].

In this regard, Vit E (fat-soluble antioxidant) and Vit C (as a potent water-soluble antioxidant) recovered the cypermethrin-induced oxidative stress probably through removing the produced free radicals [38]. Free radical scavenging ability of Vit E mitigates lipid peroxidation (LPO) leading to protect unsaturated membrane lipids from oxidation [39, 40]. Moreover, the generated free radicals can be removed by Vit C [41]. Besides this, Vit C helps Vit E reduce LPO content by recycling the oxidized form of it [42]. In line with the mentioned arguments, our results in this study emphasize that Vit E and Vit C effectively relieve the induced oxidative stress. In addition to Vits C and E, our findings showed that consumption of Vit A can also attenuate the negative effects of ZnO NPs on male reproductive organ. Similarly, Yucel et al. [43] reported that all-transretinoic acid (ATRA), known as a metabolite of Vit A, strongly mitigates the cisplatin-induced testicular dysfunction in rats. According to the present study and some others [44, 45], administration of Vit A partially prevents the induction of oxidative stress. Hence, the consumption of Vits A, C, and E could prevent the destructive effects of ZnO NPs on male reproductive organ.

## 5. Conclusion

In conclusion, our study highlights that ZnO NPs have harmful effects on the testes of Wistar rats, whereas Vit A, C, and E intake may mitigate the damage brought on by ZnO NPs. Vit intake may thus help prevent harm to the male reproductive system by lowering oxidative stress. Our research indicates that the rats given Vits E, C, and A had the strongest protective effects, respectively. However, it appears that further studies are still required to evaluate the ZnO NP-related apoptosis pathways accompanied by the protective potential of Vits on testis tissue.

## Figures and Tables

**Table 1:** Primer sequences used for qRT-PCR.

Gene	Forward	Reverse	Product size (bp)
*β*-Actin	ATCAGCAAGCAGGAGTACGAT	AAAGGGTGTAAAACGCAGCTC	94
SOD	TCAGGACAGATTACAGGATTAAC	TCATCTTGTTTCTCGTGGAC	281
GPx	GGGACTACACCGAAATGAATG	TCACTCGCACTTCTCAAAC	191
CAT	TGGTTAATGCGAATGGAGAG	ATAATCCGGGTCTTCCTGTG	116

Note: Primer3 software was applied for primer designing, and BLAST search was done to check the sequences.

**Table 2:** Testis weight and testis/body weight ratio in the control and all experimental groups.

Groups	Testis weight (g)	Testis index (mg/g)
Control 1	1.61 ± 0.04	5.55 ± 0.26
Control 2	1.58 ± 0.01	5.54 ± 0.25
Vit A	1.47 ± 0.02	5.27 ± 0.24
Vit C	1.52 ± 0.04	5.54 ± 0.16
Vit E	1.5 ± 0.05	5.52 ± 0.32
ZnO NPs	1.39 ± 0.05^∗^	5.7 ± 0.45
Vit A+ZnO	1.52 ± 0.04	5.54 ± 0.15
Vit C+ZnO	1.42 ± 0.05	5.2 ± 0.15
Vit E+ZnO	1.5 ± 0.06	5.67 ± 0.21

Notes: body weights and organ weights (absolute weights) are reported in grams. Organ weight/body weight ratios (testis index) are calculated as mg of organ weight/g body weight. Results are shown as mean ± SEM. ^∗^*p* < 0.05 compared to control.

**Table 3:** Effects of zinc oxide nanoparticles and vitamins A, C, and E treatment on sperm parameters.

Groups	Count (×10^6^/ml)	Normal morphology (%)	Motility (%)	Viability (%)
Control 1	21.5 ± 2.2	91.33 ± 1.58	79 ± 1.15	91.83 ± 2.37
Control 2	17.5 ± 1.5	94.5 ± 1.78	77.83 ± 4.33	88.5 ± 2.7
Vit A	19.5 ± 1.17	92.17 ± 2.17	78.33 ± 5	88.67 ± 3.42
Vit C	17.5 ± 2.48	89.17 ± 2.5	75.17 ± 6.09	86.5 ± 2.6
Vit E	22.17 ± 1.19	92.67 ± 1.9	73 ± 4.96	89 ± 0.9
ZnO NPs	11.42 ± 1.65^∗^	79.67 ± 2.71^∗^	55.17 ± 3.13^∗^	76.17 ± 2.1^∗^
Vit A+ZnO	18.67 ± 1.8	89.5 ± 2.5	72.33 ± 2.6	87.5 ± 1.5
Vit C+ZnO	18.33 ± 1.4	90.5 ± 2.32#	73.33 ± 4.7	85.33 ± 3
Vit E+ZnO	21 ± 2.8#	91 ± 1.23#	70.17 ± 5.62	87 ± 3.5

Notes: results are shown as mean ± SEM. ^∗^*p* < 0.05 compared to the control. ^#^*p* < 0.05 compared to the ZnO NP group.

## Data Availability

All data used and analyzed during the current study are included in this manuscript and available from the corresponding author upon reasonable request.
